# Ultradian rhythms of AKT phosphorylation and gene expression emerge in the absence of the circadian clock components *Per1* and *Per2*

**DOI:** 10.1371/journal.pbio.3001492

**Published:** 2021-12-30

**Authors:** Rona Aviram, Vaishnavi Dandavate, Gal Manella, Marina Golik, Gad Asher

**Affiliations:** Department of Biomolecular Sciences, Weizmann Institute of Science, Rehovot, Israel; National Institutes of Health, UNITED STATES

## Abstract

Rhythmicity of biological processes can be elicited either in response to environmental cycles or driven by endogenous oscillators. In mammals, the circadian clock drives about 24-hour rhythms of multitude metabolic and physiological processes in anticipation to environmental daily oscillations. Also at the intersection of environment and metabolism is the protein kinase—AKT. It conveys extracellular signals, primarily feeding-related signals, to regulate various key cellular functions. Previous studies in mice identified rhythmicity in AKT activation (pAKT) with elevated levels in the fed state. However, it is still unknown whether rhythmic AKT activation can be driven through intrinsic mechanisms. Here, we inspected temporal changes in pAKT levels both in cultured cells and animal models. In cultured cells, pAKT levels showed circadian oscillations similar to those observed in livers of wild-type mice under free-running conditions. Unexpectedly, in livers of *Per1*,*2*^*−/−*^ but not of *Bmal1*^*−/−*^ mice we detected ultradian (about 16 hours) oscillations of pAKT levels. Importantly, the liver transcriptome of *Per1*,*2*^*−/−*^ mice also showed ultradian rhythms, corresponding to pAKT rhythmicity and consisting of AKT-related genes and regulators. Overall, our findings reveal ultradian rhythms in liver gene expression and AKT phosphorylation that emerge in the absence of environmental rhythms and *Per1*,*2*^*−/−*^ genes.

## Abstract 2

This study reveals ultradian (16-hour) rhythms in the activation of the protein kinase AKT in the livers of mice, accompanied by corresponding downstream changes in gene expression. Intriguingly, these oscillations emerge in the absence of rhythmic environmental cues and in mice lacking the circadian clock proteins Per1 and Per2.

## Introduction

A fundamental facet of life on earth is exposure to a 24-hour rhythmic environment due to earth’s daily rotation. Throughout evolution, 2 distinct adaptations, which enable organisms to cope with these pervasive daily oscillations, have emerged: (i) an acute response to rhythmic environmental cues through externally driven signaling cascades; and (ii) a proactive anticipatory mechanism that relies on an intrinsic circadian clock.

In mammals, the circadian clock is present in almost every cell of the body and functions based on a network of transcription–translation feedback loops [1,2]. The heterodimer of BMAL1 and CLOCK (or its paralog NPAS2) drives the expression of *Period* (*Per1*, *Per2*, and *Per3*) and *Cryptochrome* (*Cry1* and *Cry2*) genes. In turn, PERIOD and CRYPTOCHROME proteins accumulate and repress the transcription of their own genes. An additional essential feedback loop involves the expression of the nuclear receptors NR1D1/2 and ROR, which regulate *Bmal1* transcription. These so termed “core clock components” not only interact with one another, but also orchestrate a myriad of cellular metabolic processes [3,4].

The PI3K–AKT signaling pathway relays environmental information of nutritional/metabolic state to regulate cell size and proliferation [5,6]. This signaling cascade relies on the phosphorylation of phosphatidylinositol (PI) to generate PIP, PIP2, or PIP3 (PIs with 1, 2, or 3 phosphorylated residues, respectively), which subsequently facilitate phosphorylation and activation of downstream targets. A principal effector is the serine/threonine protein kinase AKT. Binding to either PIP2 or PIP3 leads to phosphorylation of multiple sites of AKT, out of which the Serine residue 473 (pAKT) is required for its maximal activity and conventionally serves as readout for its activation [7]. Once AKT is activated, it phosphorylates dozens of target proteins that convey the signal to regulate gene expression and other key cellular functions. Overall, this pathway is widely known to be activated in response to feeding-related signals [6,7].

Indeed, previous studies that examined daily changes in AKT phosphorylation in mice fed ad libitum found elevated levels of pAKT at nighttime, when the animals normally ingest food [8]. Furthermore, time-restricted feeding protocols (i.e., daytime or nighttime) showed that feeding rhythms are sufficient to generate cycles of AKT phosphorylation [8,9]. Thus, rhythmic activation of AKT can be achieved in response to external signals such as food ingestion; however, it is still unknown whether they can be driven through intrinsic mechanisms, such as the circadian clock.

To test this, we studied temporal activation of AKT both in cultured cells and animal models. We found that pAKT exhibits cell-autonomous circadian oscillations. Similar rhythms in pAKT levels were observed in livers of wild-type mice under free-running conditions. Unexpectedly, experiments with circadian clock mutant mice uncovered ultradian rhythms (about 16 hours) of liver pAKT levels and AKT-related gene expression specifically in *Per1*,*2*^*−/−*^ mice.

## Results

### AKT exhibits cell-autonomous circadian phosphorylation rhythms in cultured cells

**Fig 1:**
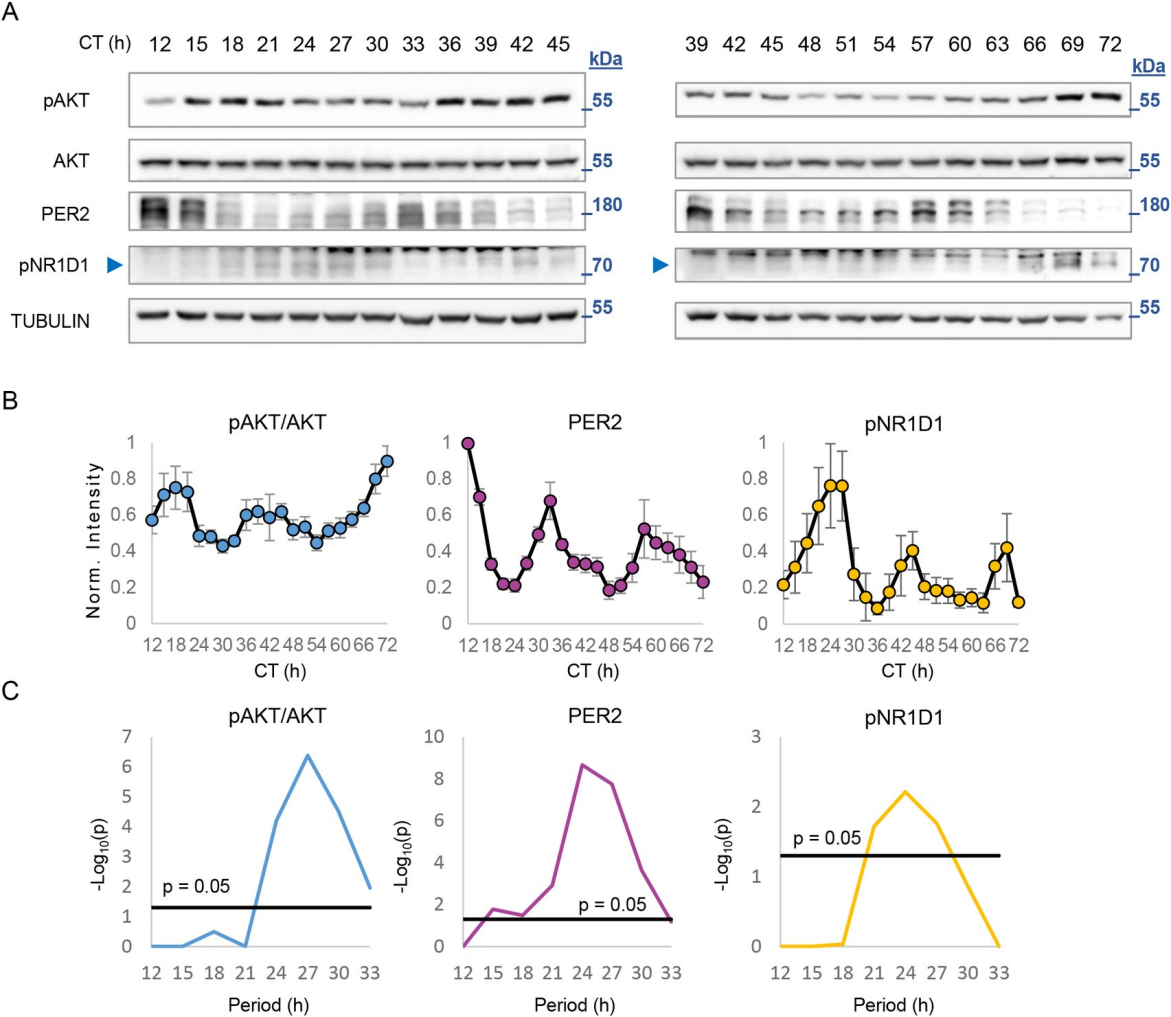
AKT exhibits cell-autonomous circadian phosphorylation rhythms. **(A)** Immunoblot analysis of protein samples from 3T3-L1 cells collected at 3-hour intervals at the indicated times. The samples for CTs 39 to 45 were run twice on 2 separate gels to enable normalization between same antibodies on different blots and quantification of the entire series as one sequence (see Materials and methods). **(B)** Intensity quantification of pAKT/AKT, PER2, and pNR1D1 protein levels. Values were normalized to the maximum for each blot (mean ± SEM, *n* = 3 to 4 technical replicates, each consists of a mixture of *n* = 3 biological replicates). **(C)** Periodogram derived from B, based on JTK_CYCLE test. CT, circadian time, time since dexamethasone shock; arrow marks the position of the specific band. The molecular mass is marked in kDa. Numerical values for panels B and C can be found in S1 Raw Data. kDa, kilodalton.

To test whether pAKT rhythmicity is endogenously driven, we examined its phosphorylation level in cell culture, a relatively constant environment. Time course analysis with cultured 3T3-L1 cells revealed rhythmic pAKT levels (Fig 1A). JTK_CYCLE analysis [10] showed that pAKT levels, similar to PER2 and pNR1D1, oscillate with a period of about 24 hours (Fig 1B and 1C). We concluded that in cultured cells, pAKT levels exhibit cell-autonomous circadian oscillations.

AKT phosphorylation was previously shown to be cell cycle regulated, with elevated activation of AKT at the G2 phase [11]. If the rhythms observed herein are driven by the cell cycle, it entails that (i) the cells in the culture are dividing; and (ii) the divisions are synchronized in coordination with AKT phosphorylation (i.e., doubling time and prevalence of G2 cells correspond to pAKT rhythmicity). The 3T3-L1 cells indeed proliferate but with a doubling time of 33 hours, beyond the observed period for pAKT (S1A Fig). This, together with propidium iodide staining (S1B Fig), demonstrated that these divisions are not synchronized and do not oscillate in coordination with AKT phosphorylation.

Collectively, these results suggested that pAKT exhibit cell-autonomous circadian oscillations that are cell cycle independent.

### AKT exhibits ultradian phosphorylation cycles in *Per1*,*2*^*−/−*^ mice under free-running conditions

**Fig 2:**
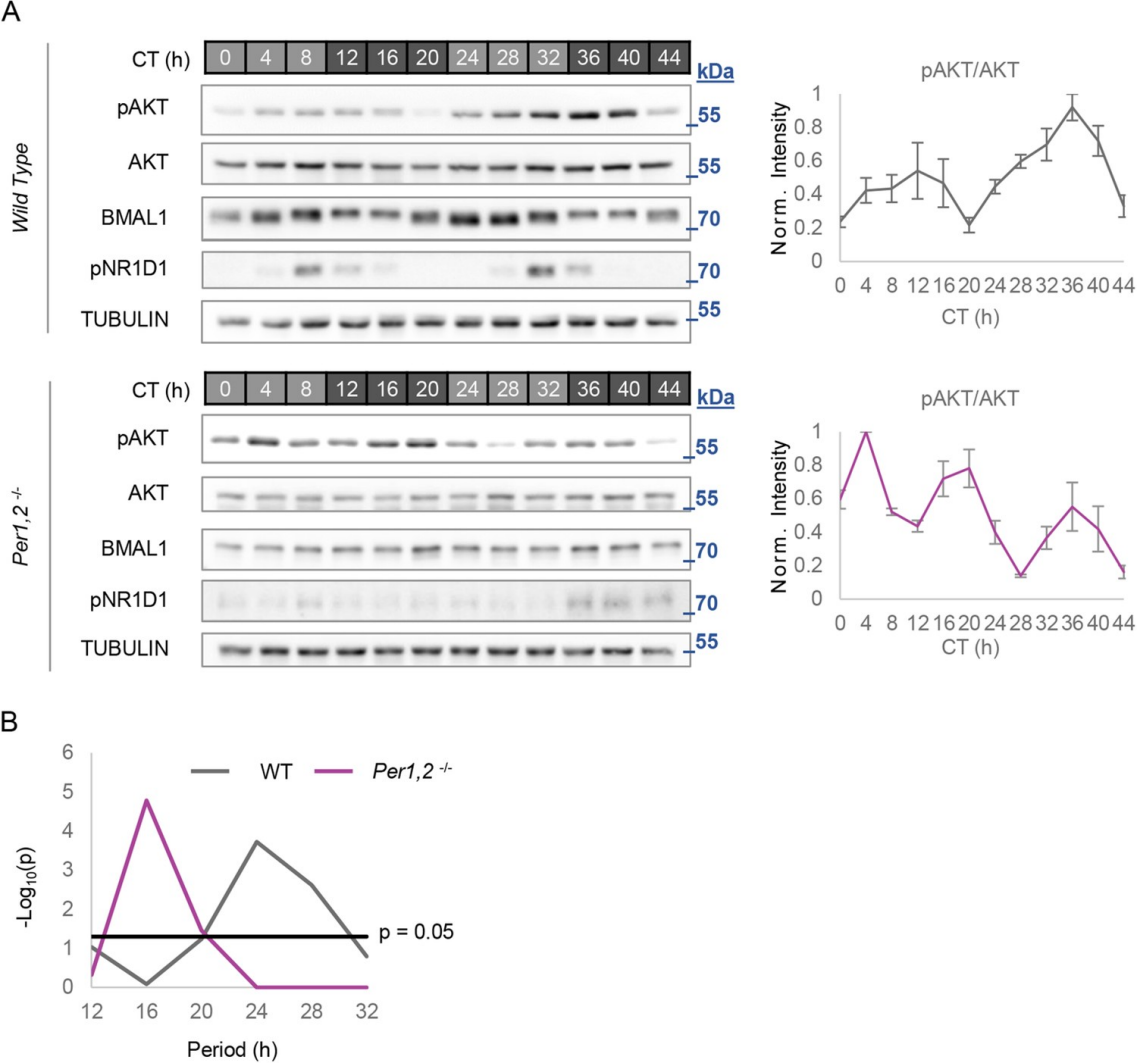
Ultradian rhythms of pAKT in *Per1*,*2*^*−/−*^ mice under free-running conditions. **(A)** Immunoblot analyses of protein samples extracted from livers of WT or *Per1*,*2*^*−/−*^ mice housed in constant dark regimen. Next to each blot an intensity quantification of pAKT/AKT. Values were normalized to the maximum for each blot (mean ± SEM, *n* = 3 to 4 mice per time point). **(B)** Periodogram derived from A, showing a dominant period of about 24 hours for WT and about 16 hours for *Per1*,*2*^*−/−*^ (JTK_CYCLE test). The molecular mass is marked in kDa. Numerical values for panels A and B can be found in S1 Raw Data. CT, circadian time; kDa, kilodalton; WT, wild-type.

Our observations in cell culture prompted us to examine whether AKT phosphorylation rhythms are also present in vivo in mice. Analysis of pAKT in liver of wild-type mice under free-running conditions (i.e., in constant dark) over the course of 2 days showed about 24-hour rhythms (Fig 2A and 2B), consistent with the animals’ rhythmic feeding behavior (S2A and S2B Fig). To eliminate any circadian effects that might mask or interfere with the endogenous pAKT rhythms, we tested clock mutant *Per1*,*2*^*−/−*^ mice [12] housed in constant dark and fed ad libitum. As expected, *Per1*,*2*^*−/−*^ mice showed no rhythmicity of clock proteins (as demonstrated by absence of BMAL1 and pNR1D1 rhythmicity) or feeding behavior (S2A and S2B Fig) (see also [12,13]). Unexpectedly, under these conditions, we detected ultradian rhythms in pAKT levels, with a period of about 16 hours (Fig 2A and 2B). This suggested that presence of ultradian pAKT oscillations, in vivo, in the absence of environmental rhythms and the circadian clock.

### Liver gene expression cycles with ultradian periodicity in *Per1*,*2*^*−/−*^ mice under free-running conditions

As a major cell signaling node, AKT has multitude downstream targets that participate in gene expression regulation [5]. To examine whether the observed ultradian AKT rhythmic activation corresponds to changes in gene expression, we performed RNA sequencing (RNA-seq) on either wild-type or *Per1*,*2*^*−/−*^ mice, housed under constant conditions (i.e., constant dark). RNA was extracted from livers collected over a 2-day time course at 4-hour intervals.

**Fig 3:**
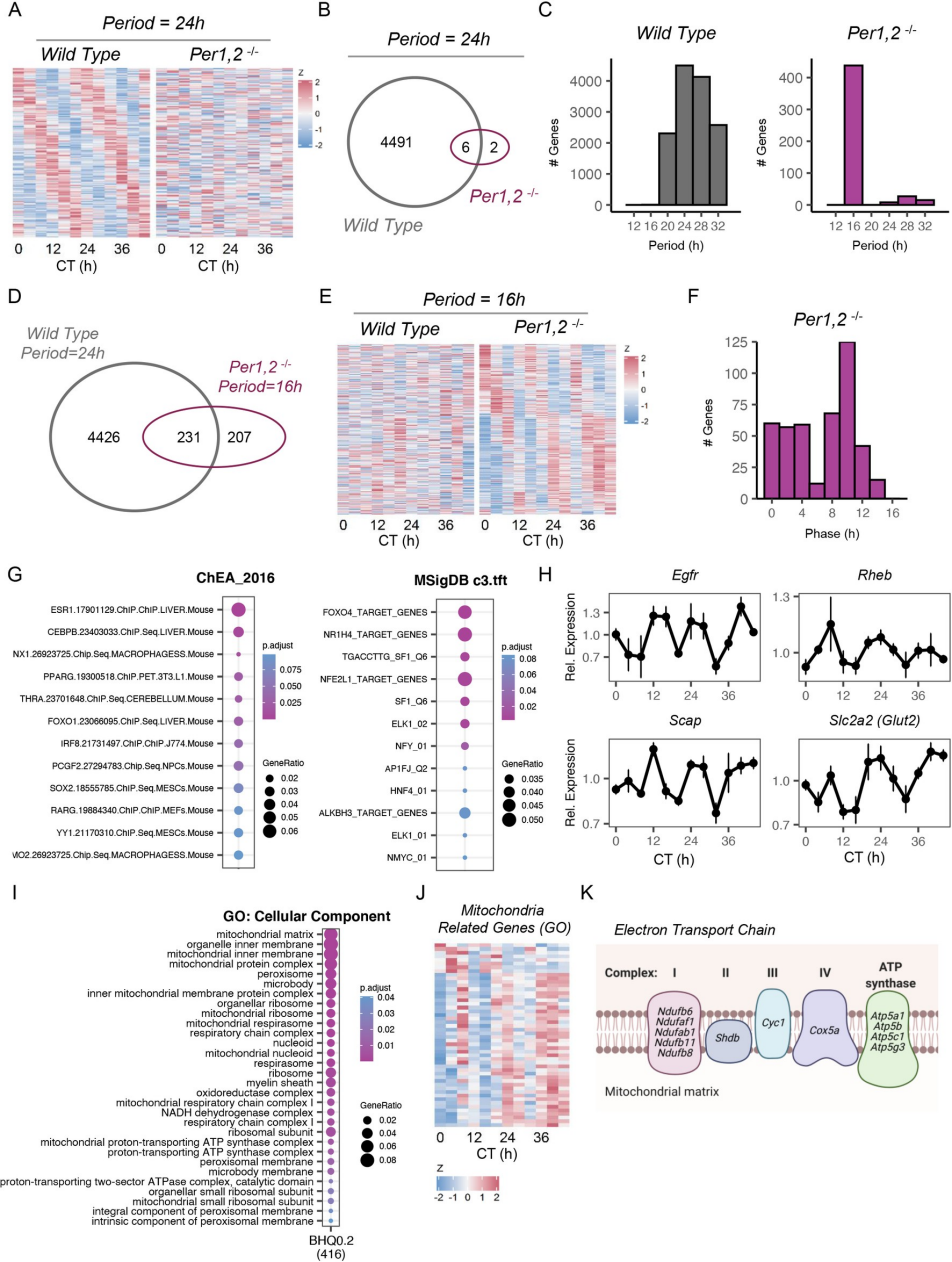
Liver gene expression cycles with ultradian periodicity in *Per1*,*2*^*−/−*^ mice under free-running conditions. **(A)** Heatmap of expression profiles of genes that were rhythmic (q < 0.2, JTK_CYCLE analysis) in WT with an about 24-hour period and their corresponding profiles in *Per1*,*2*^*−/−*^ mice. Data are presented as z-scores of the average expression in each CT. **(B)** Venn diagrams representing the overlap between 24-hour rhythmic genes in WT or *Per1*,*2*^*−/−*^ mice. **(C)** Periodograms of the transcriptome in WT or *Per1*,*2*^*−/−*^ mice. **(D)** Venn diagrams representing the overlap between 24-hour rhythmic genes in WT or 16-hour rhythmic genes in *Per1*,*2*^*−/−*^ mice. **(E)** Heatmap of expression profiles of the rhythmic genes in *Per1*,*2*^*−/−*^ mice with an about 16-hour period and their corresponding profiles in WT. Data are presented as z-scores of the average expression in each CT. **(F)** Histogram representing the distribution of phases of 16-hour rhythmic genes in *Per1*,*2*^*−/−*^ mice. **(G)** Enrichment analysis of rhythmic genes of 16-hour rhythmic genes in *Per1*,*2*^*−/−*^ mice based on ChEA dataset (left) and MSigDB C3:TFT collection (right) (*p* < 0.1, overrepresentation test). **(H)** Daily profiles of selected 16-hour rhythmic genes in *Per1*,*2*^*−/−*^ mice. **(I)** GO cellular compartment enrichment analysis of 16-hour rhythmic genes in *Per1*,*2*^*−/−*^ mice (*p* < 0.05, overrepresentation test). **(J)** Heatmap of expression profiles of mitochondria related genes that were rhythmic with about 16-hour period in *Per1*,*2*^*−/−*^ mice. Data are presented as z-scores of the average expression in each CT. **(K)** Graphic representation of mitochondrial electron transport chain complexes, highlighting related genes that were rhythmic with about 16-hour period in *Per1*,*2*^*−/−*^ mice. See also S1 and S2 Tables. CT, circadian time; GO, Gene Ontology; WT, wild-type.

Wild-type animals exhibited circadian rhythmicity in gene expression, as about 40% of the transcripts detected cycled with an about 24-hour period (according to JTK_CYCLE analysis, q < 0.2) (Fig 3A and 3B, S1 Table) [14,15]. These about 24-hour rhythms were largely eliminated in *Per1*,*2*^*−/−*^ mice, with only 8 genes deemed statistically rhythmic.

Our observation that pAKT exhibits ultradian rhythms in liver of *Per1*,*2*^*−/−*^ mice prompted us to examine periods other than 24 hours. Strikingly, we detected rhythmicity with an about 16-hour period, which was essentially the only other period detected in this method. Similar conclusions were drawn across additional data filtration threshold, significance cutoffs, as well as other widely used rhythmicity tests (i.e., harmonic regression [16] and RAIN [17]) (S3A–S3D Fig, S1 Table).

Roughly half of the knockout 16-hour rhythmic genes were de novo oscillations (i.e., were not rhythmic in wild type), whereas the other half showed about 24-hour rhythmicity in wild-type mice (Fig 3D). Within the 48-hour window of collection, 3 peaks could be observed, with a bimodal phase distribution (Fig 3E and 3F). Their median amplitude was slightly lower than that of the wild-type 24-hour rhythmic genes (S3E Fig, S1 Table).

Next, we examined the annotated biological functions of the rhythmic genes in the clock mutant mice. Comparison to mouse ChEA dataset (compilation of about 200 published ChIP-seqs [18]) showed enrichment of targets for several transcription factors in mouse tissues (Fig 3G, S2 Table). Among them were ESR1 and PPAR gamma, which have been shown to work upstream of AKT, via modulation of insulin signaling and PTEN regulation [19–21]. Importantly, we observed enrichment for targets of FOXO1, a canonical downstream effector of the AKT pathway, whose FOXO4 isoform was also identified in the MSigDB C3:TFT collection [22], a transcription factor targets dataset (Fig 3G, S2 Table). An additional analysis of upstream regulators predicted the involvement of prominent AKT-related factors, such as RICTOR, PTEN, insulin, and again FOXO1, consistent with the rhythmicity of some notable genes related to this signaling pathway (S2 Table). Profiles of representative genes are presented in Fig 3H.

Interestingly, GO cellular component gene enrichment analysis revealed high enrichment for mitochondria related functions, chief among them were genes related to mitochondrial gene expression (ribosomal subunits) and mitochondrial respiration, with representatives from all 5-electron transport chain protein complexes (Fig 3I–3K, S2 Table). Notably, their phases were aligned, i.e., their expression coordinated, which might suggest functional significance.

In conclusion, the use of *Per1*,*2*^*−/−*^ mice enabled us to eliminate feeding rhythms that carry a prominent effect on AKT phosphorylation and exposed ultradian rhythms in pAKT and gene expression in mice. These rhythmic genes were enriched for AKT-related processes and mitochondrial function.

Next, we analyzed another clock mutant mouse model, namely *Bmal1*^*−/−*^ mice [12,23] and examined both liver pAKT levels and liver gene expression by RNA-seq, in constant dark for 2 consecutive days. Unlike *Per1*,*2*^*−/−*^ mice, in *Bmal1*^*−/−*^ mice we did not observe significant rhythms in neither pAKT levels nor in gene expression (aside of 12 genes that showed 12-hour rhythms) (S4 Fig, S3 Table). We, therefore, concluded that the observed about 16-hour ultradian rhythms in liver pAKT levels and gene expression specifically emerge in the absence of *Per1/2* and not *Bmal1*.

### Phosphorylation of AKT is not required for circadian clock rhythmicity

**Fig 4:**
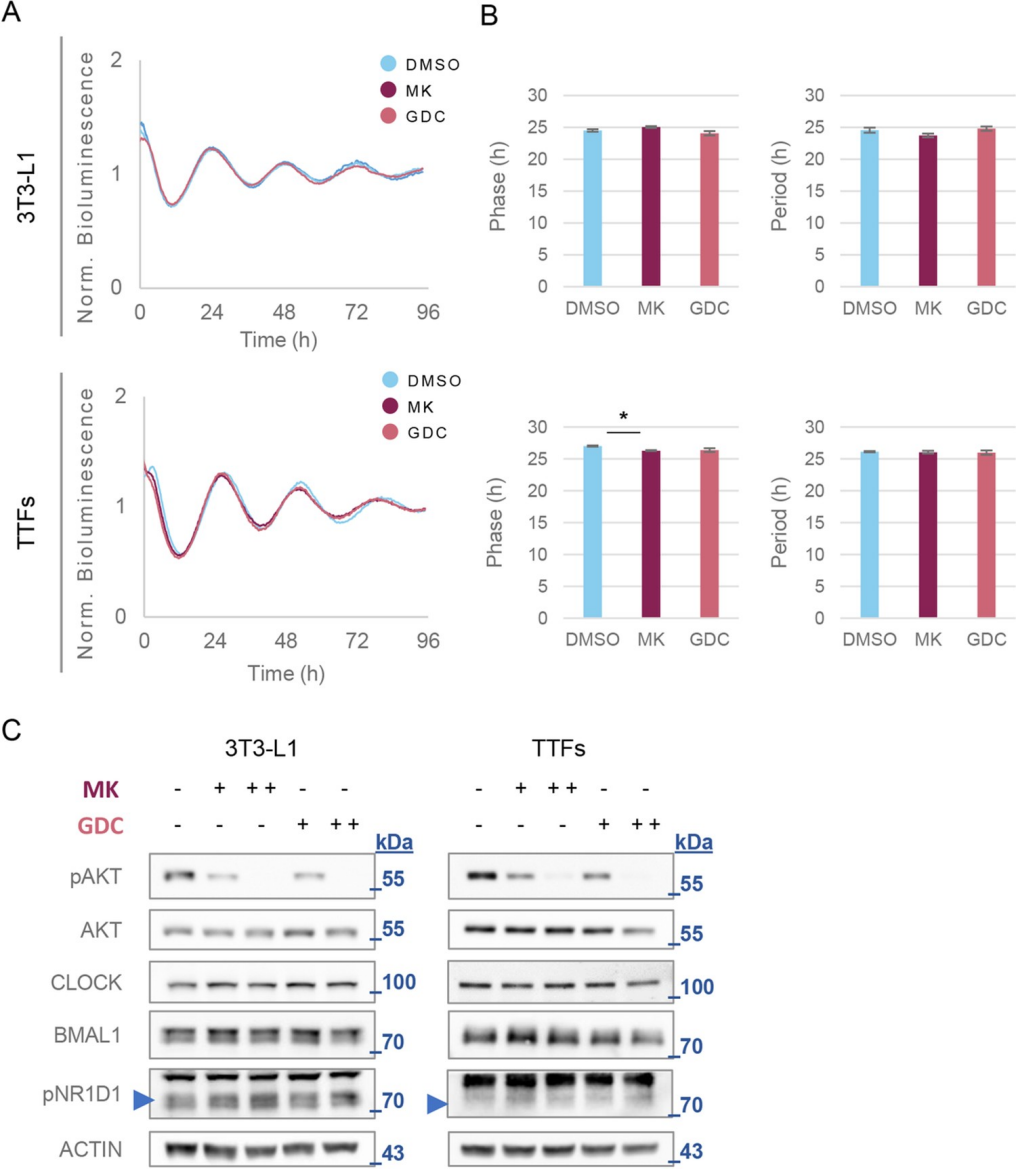
Phosphorylation of AKT is not required for circadian clock rhythmicity. **(A)** Bioluminescence recording from 3T3-L1 stably expressing a *Per2*:*luciferase* reporter (top) and TTFs prepared from PER2::LUC mice (bottom). Cells were treated with 0.2 μM of either MK-2206 (MK), GDC-0491 (GDC), or DMSO control. Data shown as mean of *n* = 3 per condition. **(B)** Analysis of the effect of MK and GDC treatment on the clock phase (as determined by the time of the first peak in A) and period (the time between the first and the second peaks) (mean ± SEM; *p < 0.05, Student *t* test). **(C)** Immunoblot analyses of protein samples from the indicated cells treated with 2 doses of MK or GDC (0.05 and 0.5 μM, + and ++, respectively) for 4 hours prior to sample collection. Arrow marks the position of the specific band. The molecular mass is marked in kDa. Numerical values for panels A and B can be found in S1 Raw Data. kDa, kilodalton; TTF, tail tip fibroblast.

Next, we examined the reciprocal relationship, namely, whether phosphorylation of AKT affects the circadian clock’s rhythmicity. Pharmacological inhibition of AKT (either directly by MK-2206 or via upstream inhibition of PI3K by GDC-0941) strongly inhibited AKT phosphorylation (S5A Fig). However, we did not observe any overt effects on the phase or period of circadian reporters; *Per2*:*luciferase* in 3T3-L1 and in tail tip fibroblasts (TTFs) from PER2::LUC mice, and overall, the clock function was unperturbed (Fig 4A and 4B). In agreement with this, the levels of several core clock proteins remained unchanged upon pAKT inhibition, across different drugs, doses, and in opposing administration times (Fig 4C, S5B Fig).

These data show that, at least in cell culture, inhibition of pAKT does not carry a prominent effect on the circadian clock function or the levels of its core proteins.

## Discussion

In this study, we found that the protein kinase AKT exhibits ultradian phosphorylation rhythms in *Per1*,*2*^*−/−*^ mouse livers that are associated with ultradian gene expression. These oscillations are intriguing as they emerge in the absence of rhythmic environmental cues (e.g., light or food intake) or known endogenous mechanisms of rhythmicity (e.g., circadian clock). Notably, these ultradian rhythms were not detected in another clock mutant mouse model, namely *Bmal1*^*−/−*^ mice, suggesting that they are not common to all clock mutant animals and raising the possibility that *Bmal1* might be implicated in these rhythms.

The molecular nature underlying this rhythmicity remains open for investigation, and at this stage, we cannot determine whether AKT is a core component of an alternative oscillator or a rhythmic output of such, nor establish a causal relation between pAKT rhythmicity and rhythmic gene expression. Negative feedback loops are a common design principle underlying oscillatory behavior [24], and in the AKT signaling pathway, at least 2 different negative feedback loops have been identified [6,25]. It is conceivable that members within this complex network, which function under certain constraints [26,27], may generate a feedback loop that drives about 16-hour rhythms. Among others, these may include lipid species such as polyphosphoinositides, primarily PIP3, which facilitate phosphorylation and activation of AKT, or proteins such as mTORC2 and RICTOR, as well as downstream AKT effectors such as phosphorylated S6K, S6, and mTOR [6,25]. Another interesting candidate is GSK3β, a target of AKT, which was recently suggested to function also as a cryptochrome-independent component of a cytosolic oscillator [28]. Furthermore, our bioinformatic analysis hints toward some transcription factor complexes, whose relevant transcripts exhibit about 16-hour rhythmicity. For instance, aside from FOXO, which functions downstream to AKT activation, other transcriptional regulators of metabolism such as PPAR and HNF might be of relevance. Future studies utilizing different AKT mutants, or kinase activity modifiers, might shed light on the molecular mechanisms underlying the ultradian rhythms observed herein. Due to the complex signaling network comprising of multiple components and feedback loops, exploring these options pose a considerable challenge. It is noteworthy that deciphering the circadian core clock machinery was achieved only decades after the groundbreaking discovery of clock mutants that differ in their circadian period length (i.e., the *Drosophila* period gene) [29–31].

If indeed pAKT is a mere output of an alternative oscillator, our finding that inhibition of AKT phosphorylation does not affect the circadian clock should be interpreted cautiously as it does not rule out a possible interaction between the oscillators. Indeed, several studies showed that other components of the PI3K–AKT pathway are implicated in the circadian clock function. Specifically, TOR was shown to affect the clock’s rhythmicity in different models such as *Arabidopsis* [32], flies (together with AKT mutants) [33], and mammals [34,35]. While neither of the pathway’s components seem to be an integral part of the mammalian clock machinery (i.e., not core clock components), they appear to be significant for determination of parameters such as period and amplitude. Furthermore, given that AKT responds to known circadian clock timing cues such as serum and oxygen [36,37], it is tempting to speculate that, while it is not required for sustaining rhythmicity, AKT phosphorylation could play a role in conveying external signals (e.g., nutritional or metabolic cues) to the circadian clock.

Over the past years, evidence from different in vitro models, from red blood cells to clock component–deficient cells, suggest the presence of circadian rhythms in the absence of the canonical clock [38–40]. For instance, it was recently shown that ex vivo liver slices and skin fibroblasts cultivated from *Bmal1* null mice exhibit about 24-hour oscillations based on several omics screens [41]. Overall, evidence point toward the involvement of metabolic cycles, redox cycles, and posttranslational processes, yet the underlying molecular mechanism(s) are largely unknown [24,39,42,43].

It is notable that these works identified about 24-hour rhythms while our study shows shorter rhythms. It appears that the period of pAKT oscillations is significantly shorter than 24 hours in livers of *Per1*,*2*^*−/−*^ mice and is within the range of 16 hours. Period estimation can be achieved through the use of different algorithms for rhythm detection (e.g., JTK_CYCLE and cosinor fitting), yet sampling resolution is critical for determining the exact period. The JTK_CYCLE methodology analyzes rhythmicity only in discrete period lengths along the sampling interval (i.e., in our case, 4-hour intervals). Consequently, it cannot distinguish between 16 hours and, for instance, 17 hours or 15 hours, but indicates that 16 hours is more likely than 12 hours or 20 hours. In addition, analysis of pAKT/AKT levels in *Per1/2* null mice using a cosinor fitting approach showed as well a period of about 16 hours (period estimate of 16.02 with 95% confidence interval of 14.88 to 17.47). It is noteworthy that a previous analysis of pAKT in liver of clock mutant *Cry1*,*2*^*−/−*^ mice showed that pAKT cycles with comparable peak times to our study [8], further supporting our findings.

Interestingly, about 16-hour rhythms in locomotor activity were reported in *Cry1*,*2*^*−/−*^ mice [28] as well as *Per1*,*2*^*−/−*^ animals under specific experimental settings [44], yet we did not detect any rhythmicity in feeding or locomotor activity of *Per1*,*2*^*−/−*^ mice in constant dark. Ultradian rhythms of gene expression were reported in mammals in vivo in harmonics of 24 hours [14]. Specifically, about 12-hour rhythms were shown in relation to endoplasmic reticulum (ER) function and the unfolded protein response [45,46]. Remarkably, these about 12-hour rhythms persisted in *Bmal1* null mice under free-running conditions [45]. The extent of about 12-hour rhythms in our *Bmal1* null mice was much lower, as only few genes showed statistically significant about 12-hour rhythms, which might stem from differences in the methods and cutoffs used for rhythm detection. Finally, ultradian rhythms were reported in other organisms among other yeast [47] and *Neurospora crassa* [48], albeit with a much shorter period, namely about 5 hours and about 7 hours, respectively. These reports, together with our findings, support the presence of a wide range of rhythms that are shorter than 24 hours. Future studies are expected to shed more mechanistic insight and uncover potential biological implications that are related to these ultradian rhythms.

## Materials and methods

### Cell culture and reagents

All cells were cultured at 37°C in a humidified incubator with 5% CO_2_. Experimental procedures were performed in high glucose DMEM (Gibco, USA) containing 100 units/mL penicillin, 100 mg/mL streptomycin, and 10% and 20% FBS for 3T3-L1 and TTFs, respectively. For time course analyses, 2.5 × 10^6^ 3T3-L1 cells were seeded in triplicates in 6-well plates and synchronized with 100 nM dexamethasone [49] after 96 hours as they reached confluency.

TTFs were cultured as previously described [50]. In short, a small piece of tail tip was minced and digested overnight in the cell incubator in DMEM supplemented with 20% FBS, 100 units/mL penicillin, 100 mg/mL streptomycin, Liberase TM Research Grade (60 ug/ml) (Roche, Switzerland), and Amphotericin B solution (1:100) (A2942, Sigma, USA). The following day, cells were washed and further expanded in the same solution (excluding the Liberase). Experiments were performed within the first 5 to 6 weeks from the tail removal, in DMEM supplemented with 20% FBS, 100 units/mL penicillin, and 100 mg/mL streptomycin.

### Protein extraction, SDS-PAGE, and immunoblot

Tissues or cells were snap frozen in liquid nitrogen immediately after dissection or upon collection and stored at −80°C until use. Tissues were homogenized by a Bead-Ruptor 24e (Omni International, USA) in the extraction buffer. Protein samples from drug-treated cells in Fig 4 and S4 Fig were extracted in RIPA buffer (150 mM NaCl, 1% NP-40, 0.5% Na-deoxycholate, 0.1% SDS, 50 mM Tris-Hcl pH 8, and 1 mM dithiothreitol), supplemented with protease inhibitors cocktail 3 (Millipore, USA), phosphatase inhibitor cocktail 3 (Sigma, USA), PMSF (1:200), Vanadate (1:500), and NaF (1:1,000). Protein concentrations were determined using BCA assay kit (Thermo Fisher Scientific).

All samples were heated at 95°C for 5 minutes in Laemmli sample buffer and analyzed by SDS-PAGE and immunoblot according to standard procedures.

Following the transfer, stage membranes were sliced according to molecular mass to enable blotting of several antibodies on the same run.

The following antibodies were used: Cell Signaling: AKT (PAN) (#2920), pAKT S473 (#4060), and pNR1D1 (#2129); Sigma: ACTIN (A3853) and TUBULIN (T9026); and custom made: BMAL1, CLOCK, and PER2 [51].

For the time course experiments in Fig 1, the samples from circadian times (CTs) 39 to 45 were run twice on 2 separate gels to enable normalization between same antibodies on different blots and quantification of the entire series as one sequence (see next section). Complete immunoblots are found in S1 Raw Images.

### Immunoblot quantification and rhythmicity analysis

Quantifications of band intensity were done using Fiji software [52]. Each blot was background subtracted using 3 locations in a signal-free area of the membrane. Next, band quantifications were normalized to the blot’s average. When relevant, each blot was normalized to its own CTs 39 to 45 average, and for rhythmicity analysis and presentation, we used CTs 39 to 45 of the first day, which were virtually identical between the 2 days following the normalization.

Rhythmicity was assessed using JTK_CYCLE (“MetaCycle” R package) [53], with default settings. Analyses were performed for each period length separately. To compare between technical replicates, each sequence was normalized to its own maximum value.

In addition, to validate the ultradian rhythmicity of pAKT in *Per1*,*2*^*−/−*^ mice, we used nonlinear least square approach, as implemented in the R function nls(), fitting the quantification data with a 4-parameter model of the form

Y = Mag + Amp*cos((t + Phase)*2*pi/Period), where Mag is the oscillation magnitude, Amp is the oscillation amplitude, and t is the time in hours. We used the following additional settings:

start = list(Mag = 0,Amp = 1,Period = 24,Phase = 0),

lower = list(Mag = 0,Amp = 0, Period = 12,Phase = -20),

upper = list(Period = 40,Phase = 20), algorithm = "port")

The 95% confidence intervals for the period was retrieved.

### Cell density measurements

Cell density was measured using an automated cell counter (Bio-Rad, USA). Cells were resuspended in 1ml Trypsin and stained with Trypan blue, according to the manufacturer’s instructions. Doubling time was calculated from the exponential fit.

### Flow cytometry

At the indicated times, cells were washed in PBS, resuspended with trypsin A, washed again, and fixed using ice-cold 70% ethanol. Staining was done for all samples simultaneously using a PBS solution with freshly diluted RNAse (50 ug/ml) and propidium iodide (25 ug/ml). For each time point, *n* = 3 were collected separately and of each plate 10,000 to 30,000 cells were measured. Gating of the cell cycle phases was determined manually and collectively for all time points, based on the staining values (intensity of the cells in G2/M as approximately twice as bright as cells in G1, S-phase cells within this range).

### Pharmacological treatments

Drugs used were Mitomycin C (M4287, Sigma, USA), MK-2066 (AdooQ), and GDC-0941 (Cayman), according to the manufacturer’s instructions, in a DMSO background which served as control. Information regarding concentrations is specified in the figure legends.

### Animals

Three months old male of the following backgrounds were used: C57BL/6 wild-type mice, *Per1*,*2*^*−/−*^ [12], and *Bmal1*^*−/−*^ mice (Jackson Laboratory, USA, B6.129-Arntl^tm1Bra^/J) [23]. TTFs for bioluminescence recordings were obtained from PER2::LUC mice [54]. Animals were housed in an SPF animal facility, at ambient temperature of about 22°C, under a 12-hour light–dark regimen and fed ad libitum. Experiments in constant dark were performed following a minimum of 14 days 12-hour light–dark regimen. ZT0 corresponded to the time lights were turned on, and ZT12 to the time lights were turned off in the animal facility. Animals were euthanized with CO_2_.

All animal experiments and procedures were conducted in conformity with and approval of the Weizmann Institute Animal Care and Use Committee (IACUC) guidelines, working within the anti-cruelty law (experiments on animals) of 1994 as stated by the Ministry of Health of the Israeli Parliament. Experiments were done in accordance with these specific applications: 05730621–1 (*Per1*,*2*^*−/−*^ mice), 08081021–1 (*Bmal1*^*−/−*^ mice), and 12340319–2 (PER2::LUC mice).

### RNA extraction

Tissues were snap frozen in liquid nitrogen immediately after dissection and stored at −80°C until used. For RNA extraction, the tissues were soaked in TRI-reagent (Sigma) and were homogenized in Bead Ruptor24e (Omni International, USA) with stainless steel beads and then proceeded by a standard TRI reagent–based RNA extraction protocol. RNA concentration was determined using NanoDrop2000 Spectrophotometer (Thermo Fisher Scientific, USA). RNA quality was validated using 2200 TapeStation (Agilent, USA).

### MARS-seq library preparation, sequencing, and processing

RNA-seq was performed as by MARS-seq as described in [55]. The bulk MARS-seq libraries were sequenced with high-output 75-bp kits (FC-404-2005, Illumina, USA) on NextSeq 500/550 Illumina sequencer.

Processing of raw sequencing data into read counts was performed via the User-friendly Transcriptome Analysis Pipeline (UTAP) [56]. Reads were trimmed using cutadapt [57] and mapped to genome (/shareDB/iGenomes/Mus_musculus/UCSC/mm10/Sequence/STAR_index) using STAR [58] (default parameters). The pipeline quantifies genes annotated in RefSeq (that have expanded with 1,000 bases toward 5′ edge and 100 bases toward 3′ bases). Counting (UMI counts) was done using HTSeq-count in union mode [59]. Count normalization was performed using DESeq2 [60] with the following parameters: betaPrior = True, cooksCutoff = FALSE, and independentFiltering = FALSE.

RNA-seq data are available from the GEO database (accession number GSE171975). All other data that support the findings of this study are available from the corresponding author upon request.

### RNA-seq statistical analysis

Genes with null reads in any of the samples were filtered out. Following this, only genes detected in both wild-type and *Per1*,*2*^*−/−*^ datasets were retained. Rhythmicity was assessed using JTK_CYCLE (“MetaCycle” R package) [53], RAIN (“rain” R package, adjp.method = "BH"), [17] and harmonic regression test (“HarmonicRegression” R package) [16], with default settings. Analyses were performed for each period length separately. Amplitudes were calculated as fold change between maximum and minimum values of the cosine fit as produced by harmonic regression. Rhythmicity analyses results for *Per1*,*2*^*−/−*^ can be found in S1 Table.

GO cellular component enrichment tests were performed using the ClusterProfiler R package [61]. Gene lists were first converted from SYMBOL to EntrezID. Overrepresentation of GO terms [62,63] was tested with default settings.

Transcription factor analysis was done based on (a) ChEA dataset [18] (ChEA_2016.csv as retrieved from https://amp.pharm.mssm.edu/Enrichr/#stats) using mouse datasets exclusively; and (b) MSigDB C3:TFT collection [22]. Conversion to human data was converted from mouse to human using biomaRt [64,65]. “Upstream regulators” analysis was performed using the Ingenuity Pathway Analysis (IPA) software (Qiagen, Germany) with default settings. Enrichment tests results can be found in S2 Table.

Analyses for *Bmal1*^*−/−*^ mice were done as described above, and wild-type samples were reanalyzed together with these samples for presentation in S4 Fig. Rhythmicity analyses results can be found in S3 Table.

### Bioluminescence recording

Cells used were TTFs from PER2::LUC mice [54] and 3T3-L1 expressing a *Per2*:*luciferase* reporter [66]. Cells were grown in a 3.5-cm culture dish with 3 ml of culture buffer, supplemented with 100 nM D-Luciferin (Promega, USA), and bioluminescence was recorded continuously for at least 4 consecutive days with LumiCycle32 recorder (Actimetrics, USA). Bioluminescence data were extracted using the LumiCycle Analysis software (Actimetrics, USA). Subsequent analyses were performed using MATLAB (MathWorks, USA). The data were detrended by normalizing it to a 48 hours moving average trend. Peaks in the data were detected by smoothing the data with a 2-hour moving average and then applying the MATLAB “findpeaks” function with a minimal peak width of 200 minutes. The first peak was considered as the phase marker, and the period was calculated as the delta between that peak and the following one.

### Statistics

All the statistical analysis was performed by either Excel, R 3.5.1, or MATLAB R2017b. Specific information on sample sizes, statistical significance, and variance measures is provided in the relevant figure legends.

## Supporting information

S1 Fig Cell cycle analysis. **(A)** Time course analysis of cell density. DT was calculated from the exponential growth slope (gray line). Shown are 2 biological replicates for each time point. **(B)** Cell cycle analysis using flow cytometry. Propidium iodide stain was used to determine the fraction of cells in G0/G1, S, and G2/M phases. Mean ± SEM, *n* = 3 biological replicates. Numerical values can be found in S1 Raw Data. DT, doubling time.
(TIF)

S2 Fig Feeding behavior analysis of WT and *Per1*,*2*^*−/−*^ mice. Reanalysis of data from Adamovich and colleagues (2019): WT or *Per1*,*2*^*−/−*^ mice were housed in metabolic cages in constant dark fed ad libitum, and their food consumption was continuously monitored. **(A)** Data are presented as moving average of a 4-hour window, with 4 mice (m, numbered 1 to 4) per condition. CT0 = the beginning of the respective light phase. **(B)** Periodogram of food consumption within a 4-hour window (JTK_CYCLE test). Numerical values can be found in S1 Raw Data. WT, wild-type.
(TIF)

S3 Fig *Per1*,*2*^*−/−*^ mice liver gene expression cycles with ultradian periodicity under free-running conditions. Periodograms of the transcriptome in WT or *Per1*,*2*^*−/−*^ mice with **(A)** different filtration method; here, only genes with at least 2 reads in at least half of the samples in each condition were included with q values below 0.2; **(B)** different significance cutoffs, as indicated. q values are based on JTK_CYCLE analysis (“BH.Q”); and **(C)** based on additional rhythmicity tests: harmonic regression and RAIN (“qvals” and “pVal”, respectively). **(D)** Venn diagrams representing the overlap between 16-hour rhythmic genes in *Per1*,*2*^*−/−*^ mice, according to JTK_CYCLE (JTK), RAIN, and harmonic regression (HR). **(E)** Amplitude distribution among the 2 genotypes. Amplitude was calculated as fold change between maximum and minimum values of the cosine fit as produced by harmonic regression. See also S1 and S2 Tables. WT, wild-type.
(TIF)

S4 Fig Liver protein levels and gene expression in *Bmal1*^*−/−*^ mice under free-running conditions. **(A)** Immunoblot analyses of the indicated proteins in liver protein extracts of *Bmal1*^*−/−*^ mice, housed in constant dark. **(B)** Intensity quantification of pAKT/AKT. Values were normalized to the maximum for each blot (mean ± SEM, *n* = 3 to 4 mice per time point). **(C)** Periodogram derived from B (q < 0.2, JTK_CYCLE analysis). **(D)** Periodograms of the transcriptome in *Bmal1*^*−/−*^ mice (q < 0.2, JTK_CYCLE analysis). **(E)** Heatmap of expression profiles of genes that were rhythmic in WT with a 24-hour period and their corresponding profiles in *Per1*,*2*^*−/−*^ mice. Data are presented as z-scores of the average expression in each CT. **(F)** Heatmap of expression profiles of the rhythmic genes in *Bmal1*^*−/−*^ mice with a 12-hour period (q < 0.2, JTK_CYCLE analysis), and their corresponding profiles in WT. Data are presented as z-scores of the average expression in each CT. CT0 = the beginning of the respective light phase. The molecular mass is marked in kDa. Numerical values for panels A and B can be found in S1 Raw Data. See also S3 Table. Arrows indicate the expected position of BMAL1 and pNR1D1. kDa, kilodalton; WT, wild-type.
(TIF)

S5 Fig Inhibition of pAKT does not affect clock protein levels. **(A)** Immunoblot analyses of protein samples from the indicated cells treated with 0.2 μM of either MK-2206 (MK), GDC-0491 (GDC), or DMSO control for 24 hours prior to collection. **(B)** Immunoblot analyses of protein samples from the indicated cells treated with 2 concentrations of MK or GDC (0.05 and 0.5μM, + and ++, respectively) for 16 hours prior to sample collection (the opposing time from the results presented in Fig 4C). Arrow marks the position of the specific band. The molecular mass is marked in kDa. kDa, kilodalton.
(TIF)

S1 Raw Images The file contains original complete immunoblots with marker overlay. Following the transfer stage membranes were sliced according to the molecular mass to enable, as much as possible, blotting of several antibodies on the same run. The experimental samples, loading order, and molecular mass markers are indicated.
(PDF)

S1 Table Transcriptomics rhythmicity analyses for WT and *Per1*,*2*^*−/−*^ mouse livers. The file contains 3 tabs **(A–C)**. Output files from JTK_CYCLE (A), harmonic regression (B), and RAIN (C) analyses. Tab B includes the Calculated Amplitude of each gene (calculated as fold change between maximum and minimum values of the cosine fit, as produced by this algorithm). See details in Methods section. WT, wild-type.
(XLSX)

S2 Table Transcriptomics enrichment analyses for *Per1*,*2*^*−/−*^ mouse livers. The file contains 4 tabs **(A–D)**. Enrichment tests presented are the supporting tables for Fig 3G and 3I: Mouse ChEA dataset (A), MSigDB C3:TFT collection (B), and GO cellular component (C). The rows in tab C correspond to datasets, transcription factors, or terms significantly enriched in the *Per1*,*2*^*−/−*^ liver transcriptomics. “GeneRatio”—the pathway members out of the experimental group; “BgRatio”—the total pathway size out of the reference genome size; “pvalue”—the overrepresentation test p-value; “qvalue”—the FDR of the p-value; “geneID”—the pathway members present in the experimental group; “Count”—the number of pathway members present in the experimental group. In addition, we included ingenuity upstream regulators analysis (D). Rows correspond to predicted upstream regulators in each category. “p-value of overlap”—the overrepresentation test p-value; “Molecule Type”—the type of upstream molecule (e.g., transcriptional regulator, receptor, etc.); “Target molecules in dataset”—the least of targets included in the gene category.
(XLSX)

S3 Table Transcriptomics rhythmicity analysis for WT and *Bmal1*^*−/−*^ mouse livers. The file contains output from JTK_CYCLE. WT, wild-type.
(XLSX)

S1 Raw Data Numerical data for graphs in figures. Each spreadsheet contains numerical data of figure panels as indicated.
(XLSX)

## Abbreviations

CT: circadian time
ER: endoplasmic reticulum
IACUC: Institute Animal Care and Use Committee
IPA: Ingenuity Pathway Analysis
PI: phosphatidylinositol
RNA-seq: RNA sequencing
TTF: tail tip fibroblast
UTAP: User-friendly Transcriptome Analysis Pipeline
