# Supplementary figures and images for: Ultradian rhythms of AKT phosphorylation and gene expression emerge in the absence of the circadian clock components Per1 and Per2
Source: PLoS Biol. 2021 Dec 30;19(12):e3001492. doi: 10.1371/journal.pbio.3001492 (PMC8718012; doi:10.1371/journal.pbio.3001492)

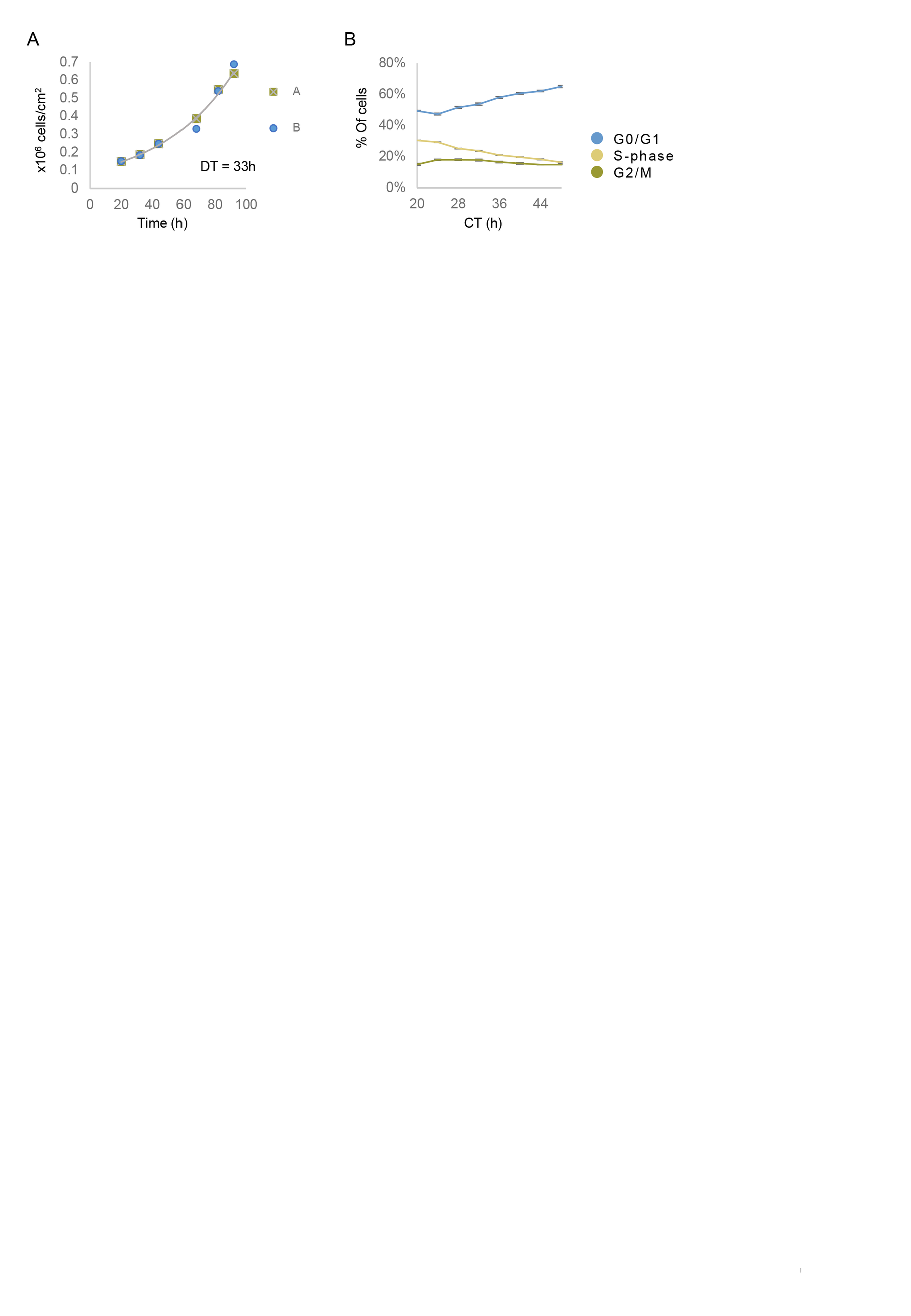

Supplement: S1 Fig — (A) Time course analysis of cell density. DT was calculated from the exponential growth slope (gray line). Shown are 2 biological replicates for each time point. (B) Cell cycle analysis using flow cytometry. Propidium iodide stain was used to determine the fraction of cells in G0/G1, S, and G2/M phases. Mean ± SEM, n = 3 biological replicates. Numerical values can be found in S1 Raw Data. DT, doubling time. (TIF) [file pbio.3001492.s001.tif]

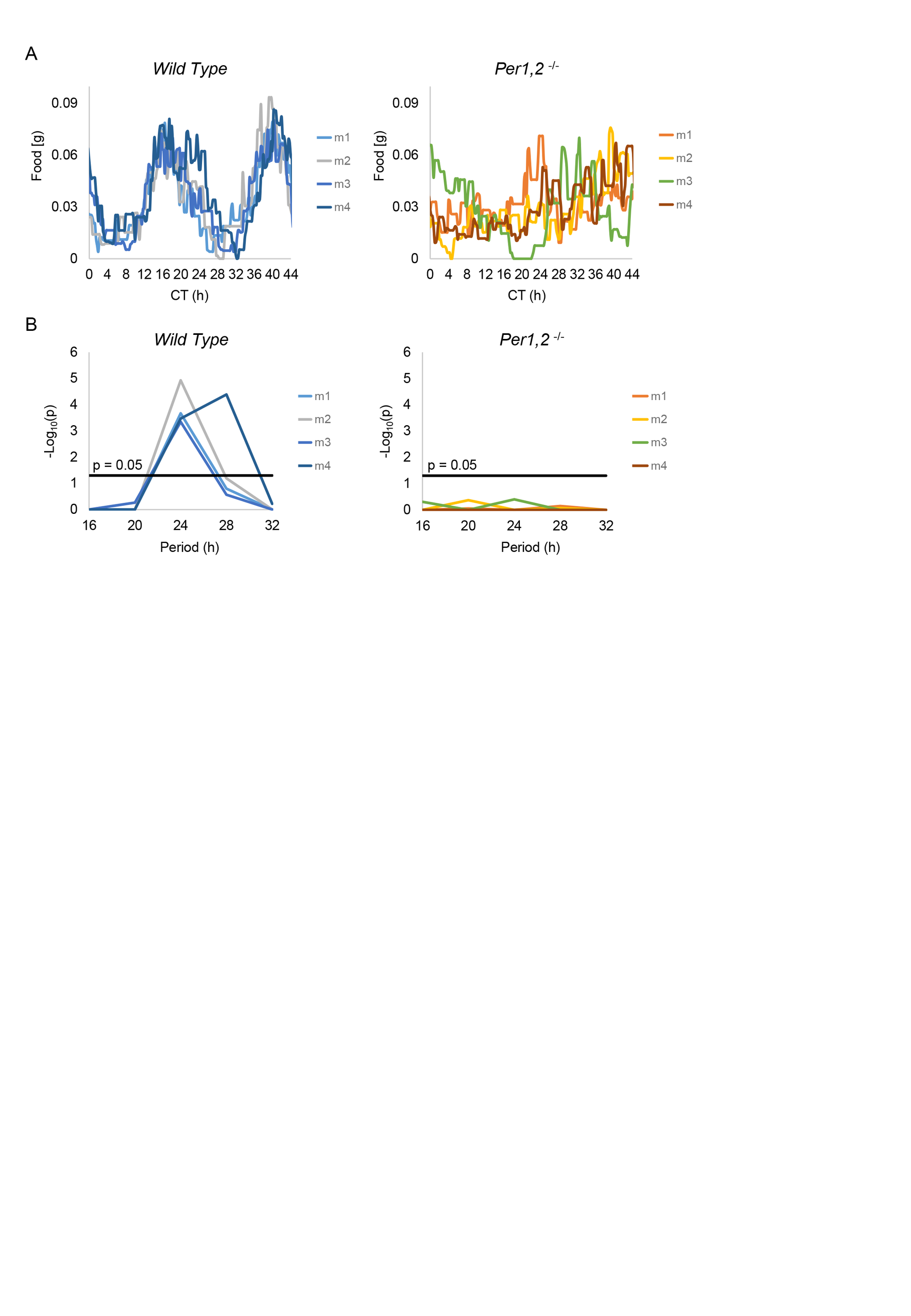

Supplement: S2 Fig — Reanalysis of data from Adamovich and colleagues (2019): WT or Per1,2−/− mice were housed in metabolic cages in constant dark fed ad libitum, and their food consumption was continuously monitored. (A) Data are presented as moving average of a 4-hour window, with 4 mice (m, numbered 1 to 4) per condition. CT0 = the beginning of the respective light phase. (B) Periodogram of food consumption within a 4-hour window (JTK_CYCLE test). Numerical values can be found in S1 Raw Data. WT, wild-type. (TIF) [file pbio.3001492.s002.tif]

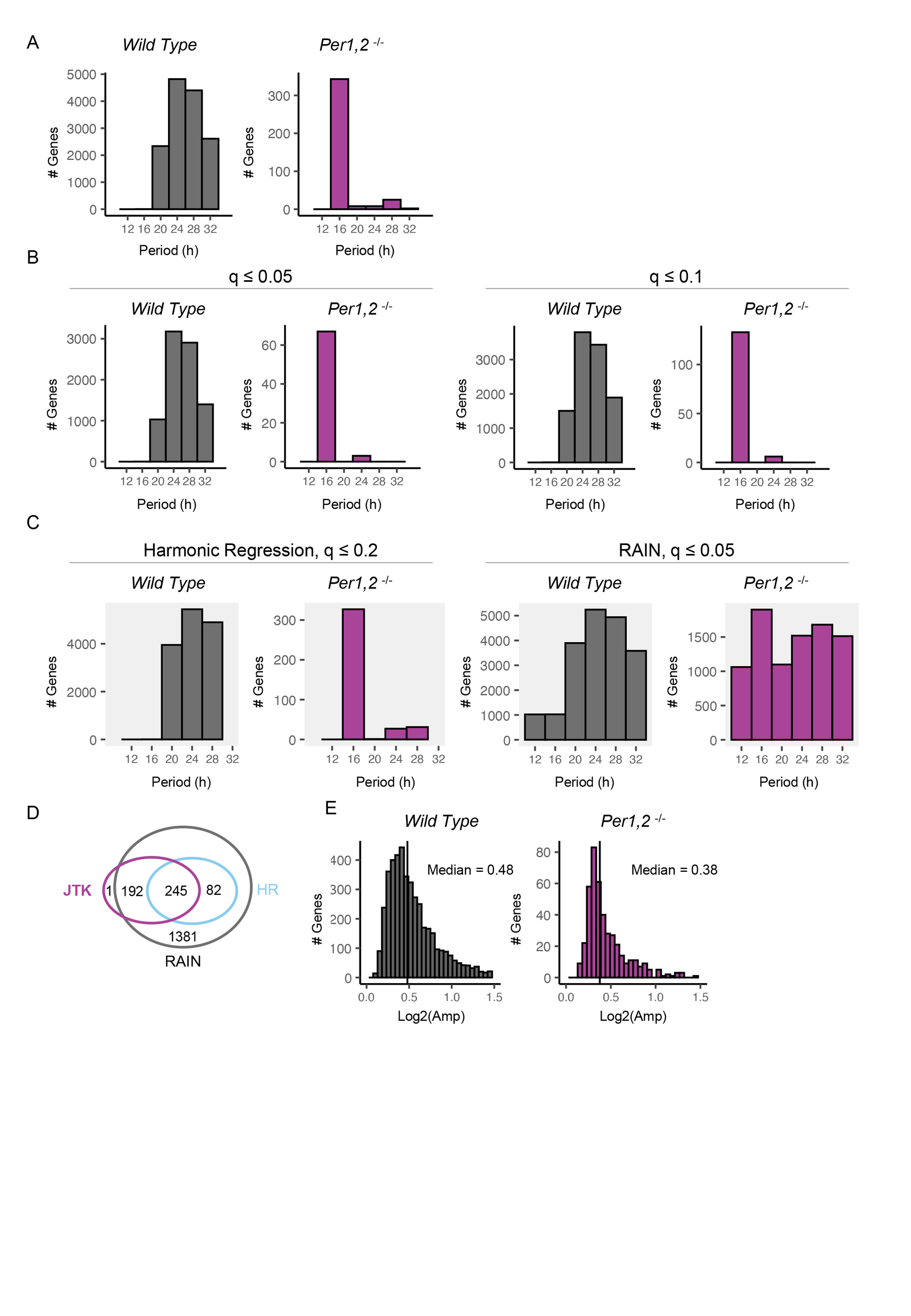

Supplement: S3 Fig — Periodograms of the transcriptome in WT or Per1,2−/− mice with (A) different filtration method; here, only genes with at least 2 reads in at least half of the samples in each condition were included with q values below 0.2; (B) different significance cutoffs, as indicated. q values are based on JTK_CYCLE analysis (“BH.Q”); and (C) based on additional rhythmicity tests: harmonic regression and RAIN (“qvals” and “pVal”, respectively). (D) Venn diagrams representing the overlap between 16-hour rhythmic genes in Per1,2−/− mice, according to JTK_CYCLE (JTK), RAIN, and harmonic regression (HR). (E) Amplitude distribution among the 2 genotypes. Amplitude was calculated as fold change between maximum and minimum values of the cosine fit as produced by harmonic regression. See also S1 and S2 Tables. WT, wild-type. (TIF) [file pbio.3001492.s003.tif]

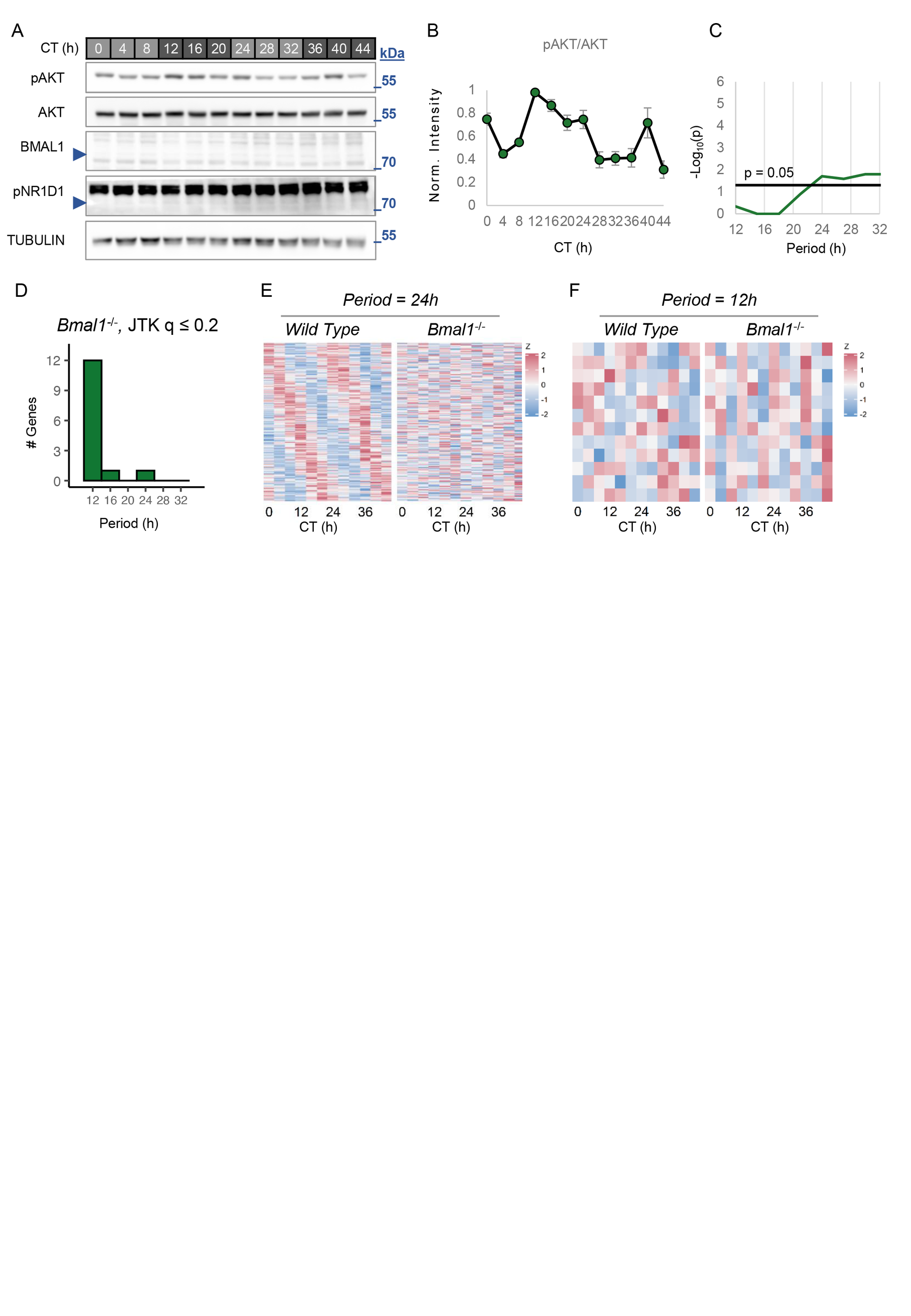

Supplement: S4 Fig — (A) Immunoblot analyses of the indicated proteins in liver protein extracts of Bmal1−/− mice, housed in constant dark. (B) Intensity quantification of pAKT/AKT. Values were normalized to the maximum for each blot (mean ± SEM, n = 3 to 4 mice per time point). (C) Periodogram derived from B (q < 0.2, JTK_CYCLE analysis). (D) Periodograms of the transcriptome in Bmal1−/− mice (q < 0.2, JTK_CYCLE analysis). (E) Heatmap of expression profiles of genes that were rhythmic in WT with a 24-hour period and their corresponding profiles in Per1,2−/− mice. Data are presented as z-scores of the average expression in each CT. (F) Heatmap of expression profiles of the rhythmic genes in Bmal1−/− mice with a 12-hour period (q < 0.2, JTK_CYCLE analysis), and their corresponding profiles in WT. Data are presented as z-scores of the average expression in each CT. CT0 = the beginning of the respective light phase. The molecular mass is marked in kDa. Numerical values for panels A and B can be found in S1 Raw Data. See also S3 Table. Arrows indicate the expected position of BMAL1 and pNR1D1. kDa, kilodalton; WT, wild-type. (TIF) [file pbio.3001492.s004.tif]

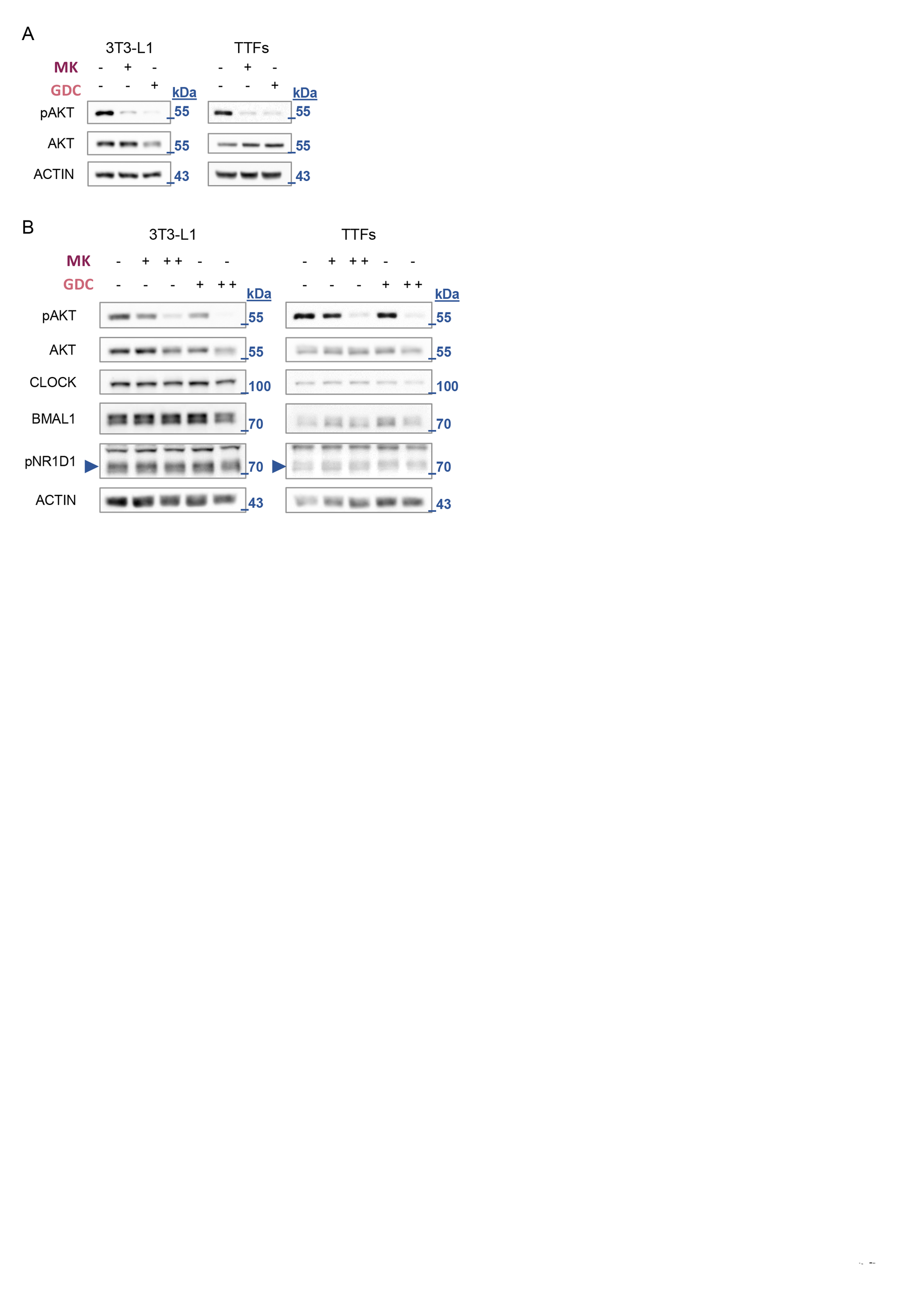

Supplement: S5 Fig — (A) Immunoblot analyses of protein samples from the indicated cells treated with 0.2 μM of either MK-2206 (MK), GDC-0491 (GDC), or DMSO control for 24 hours prior to collection. (B) Immunoblot analyses of protein samples from the indicated cells treated with 2 concentrations of MK or GDC (0.05 and 0.5μM, + and ++, respectively) for 16 hours prior to sample collection (the opposing time from the results presented in Fig 4C). Arrow marks the position of the specific band. The molecular mass is marked in kDa. kDa, kilodalton. (TIF) [file pbio.3001492.s005.tif]

Fig. 1A

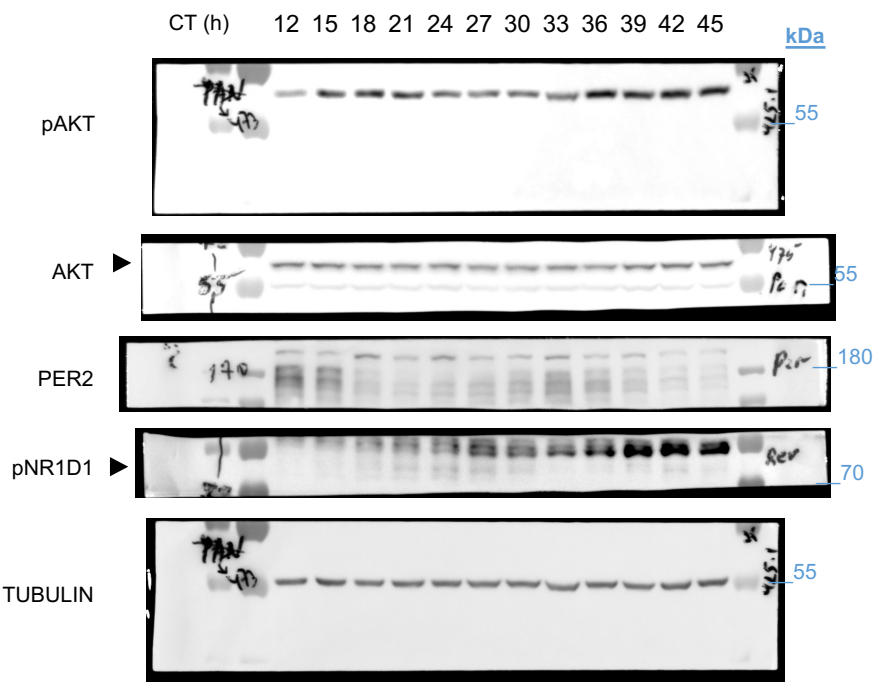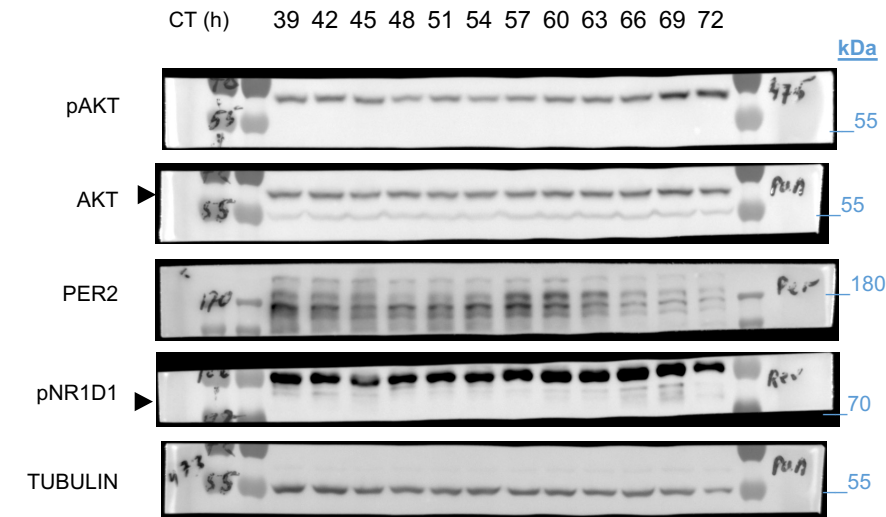

Fig. 2A

Wild Type

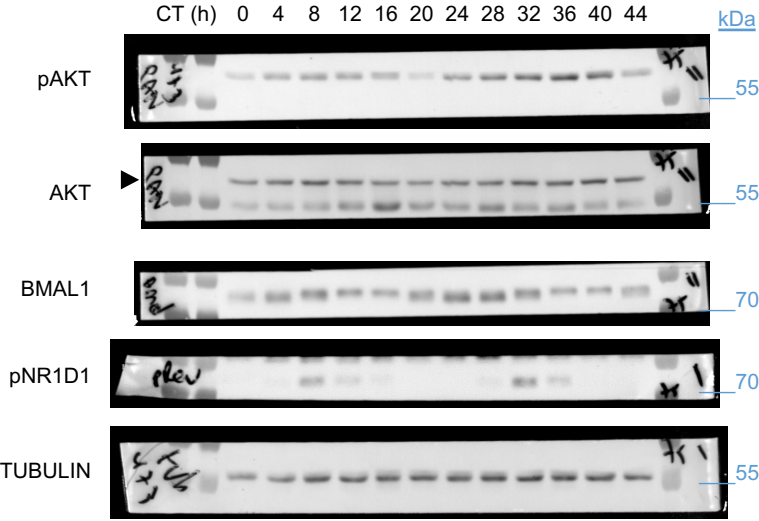

Per1,2<sup>-/-</sup>

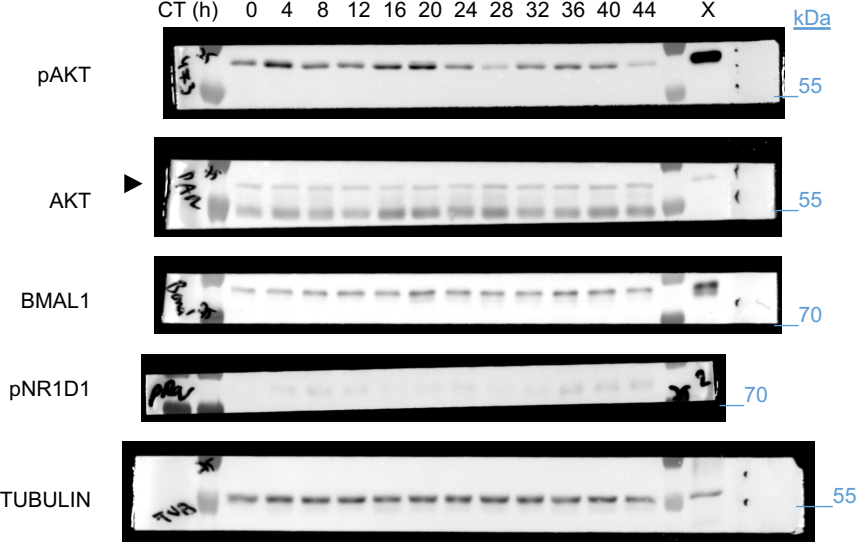

Fig. 4C

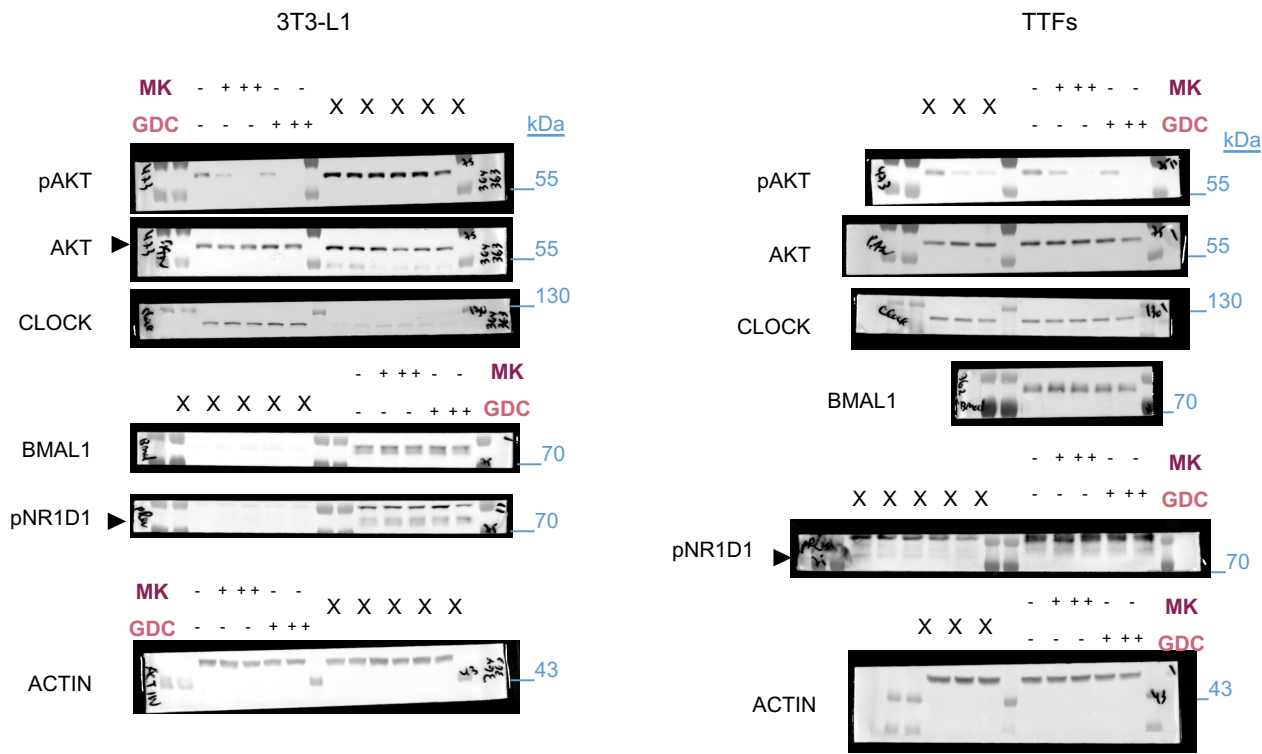

**Fig. S4A**

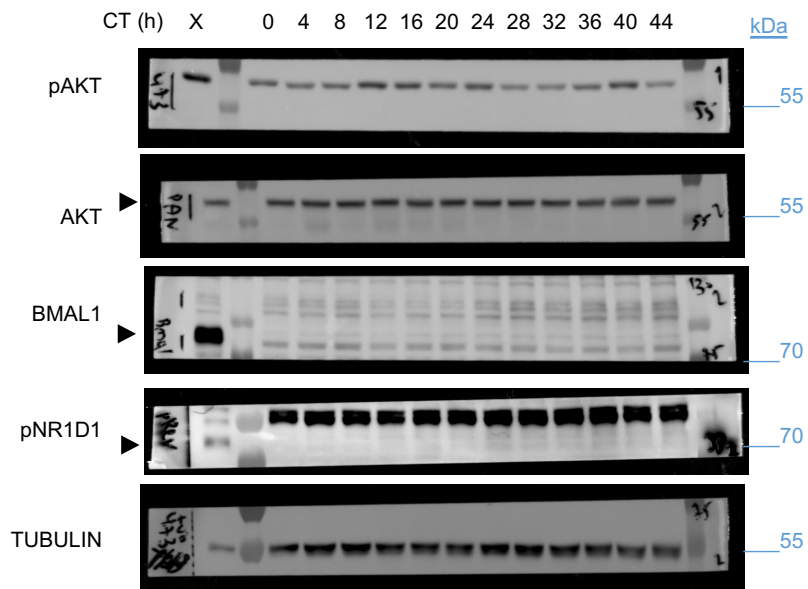

Fig. S5A

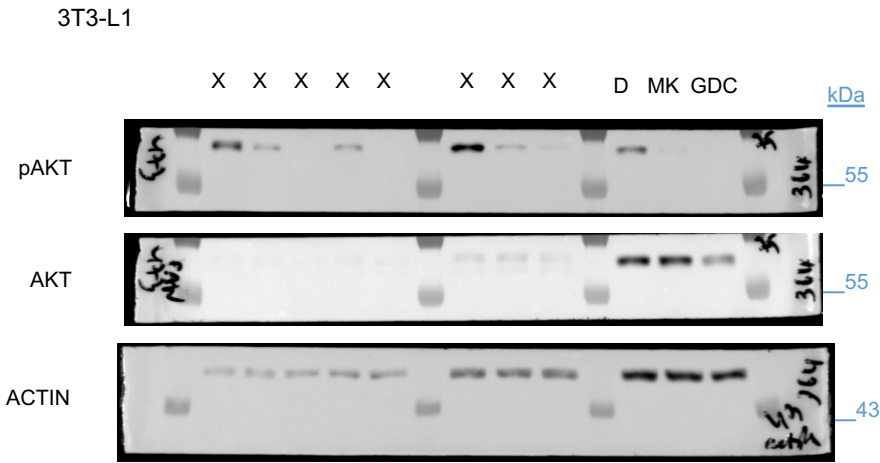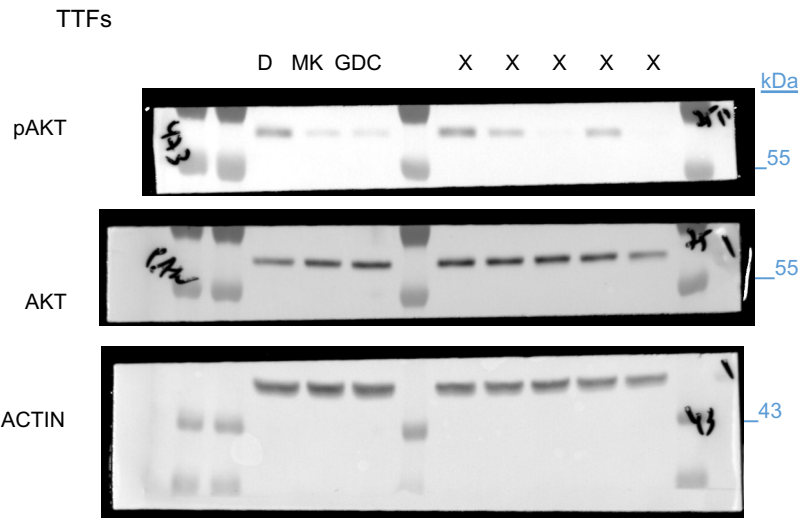

Fig. S5B

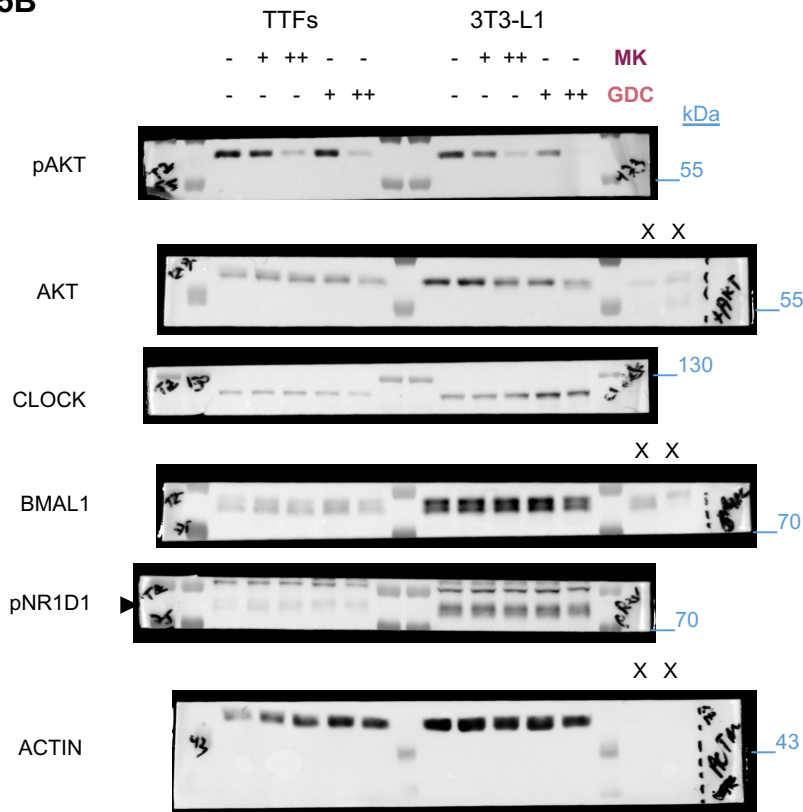

Supplement: S1 Raw Images — Following the transfer stage membranes were sliced according to the molecular mass to enable, as much as possible, blotting of several antibodies on the same run. The experimental samples, loading order, and molecular mass markers are indicated. (PDF) [file pbio.3001492.s006.pdf]
